# Debates Surrounding the Use of Antithrombotic Therapy in Hemophilic Patients with Cardiovascular Disease: Best Strategies to Minimize Severe Bleeding Risk

**DOI:** 10.3390/ijms25147845

**Published:** 2024-07-18

**Authors:** Oana-Viola Badulescu, Dragos Viorel Scripcariu, Minerva Codruta Badescu, Manuela Ciocoiu, Maria Cristina Vladeanu, Carmen Elena Plesoianu, Andrei Bojan, Dan Iliescu-Halitchi, Razvan Tudor, Bogdan Huzum, Iris Bararu Bojan

**Affiliations:** 1 Department of Pathophysiology, University of Medicine and Pharmacy Grigore T. Popa, 700115 Iasi, Romania; oana.badulescu@umfiasi.ro (O.-V.B.);; 2 Department of Surgical Sciences, University of Medicine and Pharmacy Grigore T. Popa, 700115 Iasi, Romania; 3 Department of Internal Medicine, University of Medicine and Pharmacy Grigore T. Popa, 700115 Iasi, Romania

**Keywords:** coronary artery disease, hemophilia, PCI in hemophilia, atrial fibrillation in hemophilia

## Abstract

Navigating through antithrombotic therapy in patients with both hemophilia and cardiovascular pathology presents a complex scenario with inherent challenges and opportunities. The presence of hemophilia, characterized by impaired blood clotting, adds a layer of complexity to the management of cardiovascular conditions requiring antiplatelet therapy and anticoagulation. Striking a delicate balance between the necessity for antithrombotic treatment to prevent cardiovascular events and the heightened risk of severe bleeding in individuals with hemophilia demands a nuanced and carefully considered approach. The challenges revolve around identifying an optimal therapeutic strategy that effectively mitigates cardiovascular risks without exacerbating bleeding tendencies. In hemophilic patients with cardiovascular disease, the decision to use antiplatelet therapy requires careful consideration of the individual’s bleeding risk profile, considering factors such as the severity of hemophilia, history of bleeding episodes, and concurrent medications. The goal is to provide effective antithrombotic treatment while minimizing the potential for excessive bleeding complications. Conventional anticoagulants like warfarin pose difficulties due to their potential to increase the risk of bleeding. On the other hand, emerging options like novel direct oral anticoagulants (DOACs) present an opportunity, offering predictable pharmacokinetics and user-friendly administration. However, a comprehensive exploration of their safety and efficacy in hemophilic patients is imperative. Achieving the right equilibrium between preventing cardiovascular events and minimizing bleeding risk is pivotal in selecting the most effective therapeutic option for individuals with hemophilia and cardiovascular pathology. A multidisciplinary approach, integrating the expertise of hematologists and cardiologists, becomes essential to customize treatments and address the intricacies of this medical challenge.

## 1. Introduction

Research on the role of hemostasis in ischemic cardiovascular disease (CVD) suggests that hypercoagulability increases the risk of CVD, while a bleeding tendency appears to lower it. Previous studies have shown that high levels of factor VIII are linked to a higher risk of both venous and arterial thrombosis, and many other coagulation factors have also been associated with an increased thrombotic risk. Conversely, patients with a hereditary deficiency of factor VIII (hemophilia A) or IX (hemophilia B) have a significant reduction in CVD-related mortality. A recent meta-analysis found that hemophilic patients have a 50% lower mortality rate from ischemic heart disease compared to the general population [1,2].

Two processes are necessary for an arterial thrombotic event: atherogenesis, the gradual buildup of atherosclerotic plaque, and atherothrombosis, the acute formation of an occluding thrombus. Hemostasis clearly plays a role in the formation of an occluding thrombus, and coagulation factors such as factor VIII may also contribute to atherogenesis [3,4].

It is unclear whether hemophilia protects against atherogenesis. Some studies in animals and humans suggest that factor VIII deficiency may provide protection [5,6], while others have found no such association [7]. The low incidence of cardiovascular events in hemophilic patients calls for large population-based studies. A significant limitation of previous research is the low prevalence of cardiovascular risk factors in hemophilic patients.

There are scarce studies on the prevalence of atherosclerosis in hemophilic patients with proatherosclerotic risk factors. Obesity, a major and well-established cardiovascular risk factor [8], is as prevalent among the hemophilic population as it is in the general population [9].

Even more, the life expectancy of hemophilic patients has risen from 40 years in the 1960s to 60–70 years today, except for a mortality peak in the 1980s and 1990s due to HIV infection. Many people with hemophilia now reach old age, but new challenges are emerging. Those aged 65 or older, born before the early 1940s, did not have access to regular replacement therapy for the first 25–30 years of their lives. Aging brings not only physical problems, such as CVD, but also psychological issues related to retirement and changes in family dynamics [10,11].

Since the early 1970s, the introduction of coagulation factor concentrates (first plasma-derived, then recombinant) has significantly improved the treatment of people with hemophilia (PWH), allowing for home treatment and early intervention or prevention of bleeding. Before this advancement, PWH had a life expectancy of only 20–30 years, with many dying young due to severe bleeding episodes. Since the 1990s, the widespread implementation of regular prophylaxis by hemophilia treatment centers (HTCs), which provide specialized and comprehensive care, has further enhanced clinical outcomes for PWH. Over the last decade, the development of extended half-life recombinant FVIII and FIX products, and the subcutaneous administration of the non-replacement therapy emicizumab, has improved patient adherence to prophylaxis, enabling more personalized treatment approaches. As a result, the quality of life and life expectancy for PWH have progressively increased, approaching those of the general male population in high-income countries. Consequently, hematologists are now encountering an aging population of PWH who are developing age-related conditions such as cardiovascular disease (CVD), cancers, and renal diseases [12,13]. 

## 2. Literature Search

In our comprehensive narrative review, we extensively examine the intricate interplay between antithrombotic therapy and its implications for individuals coping with both hemophilia and cardiovascular disease (CVD). We meticulously dissect the underlying pathophysiological mechanisms characterizing hemophilia and CVD, elucidating the multifaceted factors contributing to clotting imbalances. Furthermore, we emphasize the delicate equilibrium between the heightened risk of bleeding inherent in hemophilia and the complex progression of atherosclerosis observed in CVD.

We conducted a narrative review focusing on antithrombotic therapy in hemophilic patients with cardiovascular disease (CVD). A systematic search was performed on PubMed to identify relevant studies pertaining to the cardiovascular burden in hemophilic individuals, utilizing keywords such as “coronary artery disease”, “hemophilia”, “PCI in hemophilia”, and “atrial fibrillation in hemophilia”. Additionally, another PubMed search was conducted to gather significant articles elucidating the pathophysiological mechanisms underlying bleeding in hemophilic patients with CAD, employing keywords such as “percutaneous intervention”, “coronary artery bypass”, and “atrial fibrillation”. Studies were excluded if they were not in English, did not involve human subjects, or consisted of single case reports. The screening process involved three steps: reviewing titles and abstracts, assessing full manuscripts for eligibility, and selecting studies for inclusion in our review. We identified 120 results from PubMed. Studies not written in English or comprising single case reports were excluded, resulting in 61 original articles, systematic reviews and meta-analyses. Among these, one was excluded for focusing on children—see Table 1.

## 3. Cardiovascular Disease in Hemophilic Patients

**Figure 1:**
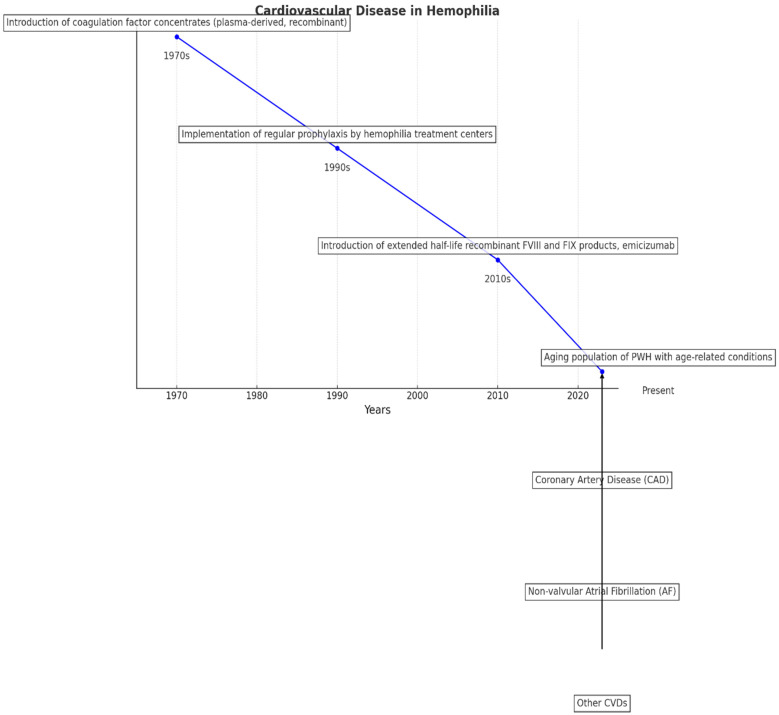
The figure highlights how advancements in treatment have led to an increased life expectancy for PWH, resulting in a growing population of older individuals with age-related health issues.

Although hemophilia is believed to offer some protection against CVD due to its inherent hypocoagulability, the exact incidence of CVD in people with hemophilia (PWH) is not well known. While several studies have shown that PWH have lower mortality from coronary artery disease (CAD) compared to the age-matched male population, recent data indicate that the prevalence of CAD in PWH increases with age. A single-center study in the USA (1993–1998) found that the age-specific prevalence of CAD ranged from 0.05% in PWH under 30 to 15.2% in those aged 60 and older [14]. Additionally, CAD-related mortality increased from 2% to 6% between 1972 and 2001, reflecting the growing life expectancy of PWH. The standardized mortality ratio (SMR) for CAD nearly doubled from 1990–1999 to 2000–2007 (0.25 to 0.55), and an SMR of 3.0 for myocardial infarction suggested a higher risk of death among PWH [15]. Despite their natural anticoagulation state, which theoretically should protect against thrombus formation, older PWH face various factors that increase their risk of developing CVD. These factors include the use of clotting factor concentrates, co-infections (such as HIV on antiretroviral therapy), hypertension, diabetes, obesity, lack of physical activity, and chronic renal disease—Figure 1 [16,17,18].

A retrospective study from Canada conducted by Minuk et al. included 294 patients from five Canadian Hemophilia Treatment Centres and identified common CVD risk factors in PWH, such as hypertension, smoking, obesity, diabetes mellitus, dyslipidemia, family history, and antiretroviral therapy, along with cardiovascular events like acute coronary syndrome, cerebrovascular ischemic disease, and atrial fibrillation (AF). Another retrospective study conducted by Sharathkumar et al. in the USA on 185 PWH found that the prevalence of CAD, stroke, and myocardial infarction was about twice as high in PWH compared to non-hemophilia males [19]. A large retrospective study in the USA that included 2506 patients with hemophilia A and 7518 in the general cohort also noted an increased prevalence of CVD in PWH compared to the general male population, while a report performed by Wang et al. on 1054 male patients with hemophilia from Taiwan showed that atherothrombotic cardiovascular events occurred at a younger age, with chronic obstructive pulmonary disease, hypertension, and hyperlipidemia being major associated risk factors [18,19]—Table 2.

## 4. Management of Coronary Artery Disease in Patients with Hemophilia

Both stable angina and acute coronary syndrome, the main types of CAD, can occur in people with hemophilia (PWH) and require the same therapeutic approaches as those without hemophilia. However, managing CAD in PWH is challenging because standard treatments involve antiplatelet and anticoagulant drugs, invasive procedures such as percutaneous coronary intervention (PCI), the use of bare metal stents (BMS) or drug-eluting stents (DES), and even coronary artery bypass grafting (CABG). Special attention is necessary due to the increased risk of bleeding associated with long-term antithrombotic treatments in PWH with CAD, as noted by the French registry [20].

### 4.1. Stable Coronary Artery Disease

In managing stable angina, long-term low-dose aspirin (≤100 mg/day) should be prescribed. For PWH with severe disease, clotting factor prophylaxis is essential to prevent worsening of the bleeding tendency. Prophylaxis is particularly important for those PWH who are ‘heavy bleeders’ or at high risk of bleeding due to comorbidities like arterial hypertension, a history of intracranial bleeding, gastrointestinal disease, liver disease, or other conditions that heighten the bleeding risk [21].

### 4.2. Acute Coronary Syndromes

Fogarty et al. found that in 15 out of 20 cases, the initial management of acute coronary syndrome (ACS) in people with hemophilia (PWH) was unchanged. When adjustments were made, they typically involved withholding or delaying aspirin or lowering the dose of low molecular weight heparin (LMWH). Hemostatic support before and after antithrombotic therapy included administering boluses of factor VIII (FVIII) or factor IX (FIX), followed by infusions, with trough levels between ≥30% and ≥50%, and/or peak levels between ≥70% and ≥100% [20,22].

A common recommendation before percutaneous coronary intervention (PCI) is to maintain peak clotting factor levels above 80% for 48 h post-procedure. The Age-Related Developments and Comorbidities in Hemophilia (ADVANCE) group recommends administering replacement therapy before PCI, aiming for peak clotting factor levels above 80%. For patients with baseline clotting factor levels of ≥25%, prophylaxis may not be required before planned PCI or coronary angiogram [22,23].

Desmopressin has been used to correct clotting factor deficiency in PWH, but it is not recommended for patients with ischemic heart disease due to its potential to increase heart rate, raise diastolic pressure, and heighten the risk of arterial thrombosis.

In an effort to standardize treatment approaches, an institutional guideline for coronary artery disease (CAD) in people with hemophilia (PWH) has been developed in the Netherlands, drawing heavily from guidelines for Dutch patients without hemophilia. More recently, an international group attempted to adapt the guidelines of the European Society of Cardiology (ESC) for managing acute coronary syndrome in PWH. By aligning these guideline recommendations with our own clinical insights, we suggest prioritizing percutaneous coronary intervention (PCI) over thrombolysis whenever feasible due to its lower risk of bleeding complications. Thrombolysis should be considered only if PCI is unavailable [10,16,24].

### 4.3. The Choice for Arterial Access in Case of Percutaneous Treatment

When considering the arterial access site, radial access is the preferred option due to its ability to lower the risk of bleeding, particularly major bleeding, making it particularly advantageous for high-risk patients such as those with hemophilia presenting with acute coronary syndrome (ACS). Past recommendations have advised against using femoral arterial access in hemophilia patients due to its elevated bleeding risk compared to radial access. However, there has been inconsistency among studies regarding the selection of arterial access sites and subsequent bleeding outcomes. For instance, Fogarty et al. found that in seven patients undergoing percutaneous coronary intervention (PCI), all but one had femoral access utilized, while Fefer et al. reported a case series of three ACS patients, all of whom underwent radial access procedures [20,21,22,23].

Given the mortality reduction associated with radial access in ST-elevation myocardial infarction (STEMI) patients, along with its decreased risk of access site bleeding and easier bleeding control, radial access has been suggested as the optimal choice in patients with hemophilia. Despite this recommendation, several cases have reported successful outcomes using femoral access with minimal complications or bleeding from the access site. Schutgens et al. advocate for radial access in their institutional guidelines, noting that up to 70% of major bleeding complications, including access site hematomas and retroperitoneal bleeds, are attributed to femoral access [18,21,25,26].

### 4.4. What Are the Best Type of Stents for PWH?

In terms of stent selection, bare metal stents (BMS) have traditionally been favored due to their shorter required duration of combined antiplatelet therapy, typically lasting one month. Although first-generation drug-eluting stents (DES) have been used in people with hemophilia (PWH), these devices take longer to endotheliaze and therefore necessitate prolonged dual antiplatelet therapy. Consequently, the main recommendation has been to use BMS along with short-term combined antiplatelet treatment.

However, second-generation DES have shown clear superiority over both BMS and earlier DES versions in terms of efficacy and safety. Furthermore, with the latest available DES, one month of dual antiplatelet therapy has proven to be as effective as longer treatment durations, especially in patients at high risk of bleeding. Therefore, our preference is to employ second-generation DES and administer one month of dual antiplatelet therapy in PWH undergoing percutaneous coronary intervention (PCI) [27,28,29].

In the study led by Fogarty et al., six patients were treated with bare metal stents (BMS), while one patient underwent implantation of a drug-eluting stent (DES). Fefer et al. reported that all three cases received BMS, with one patient experiencing in-stent restenosis after one year and subsequently undergoing cutting balloon angioplasty without encountering further complications [20]. Preference is given to using BMS with reduced thrombogenic risk, such as titanium-nitric oxide-coated stents, carbofilm-coated stents, or endothelial progenitor cell capture stents, whenever they are available. In patients with a residual clotting factor level of 25% or higher, DES is considered a viable option. With advancements in DES technology and the reduced need for prolonged antiplatelet therapy, this recommendation may be broadening. However, akin to the general population, the risk of restenosis persists, with patients showing restenosis rates of 50–70% as early as 6–12 months post PCI when not on dual antiplatelet therapy (DAPT).

### 4.5. The Choice for Antithrombotic Treatment

Antithrombotic treatment in coronary artery disease (CAD) primarily targets the inhibition of platelet aggregation and coagulation pathways to prevent the formation of thrombi, which can occlude coronary arteries and lead to myocardial infarction. Platelet inhibitors such as aspirin and P2Y12 inhibitors (e.g., clopidogrel, ticagrelor) are cornerstone therapies. Aspirin inhibits the cyclooxygenase-1 (COX-1) enzyme, reducing thromboxane A2 production, a potent promoter of platelet aggregation. It exerts its molecular effects on endothelial biology through multiple mechanisms, primarily centered around the inhibition of cyclooxygenase (COX) enzymes. Aspirin irreversibly acetylates COX-1 and COX-2, preventing the conversion of arachidonic acid into prostaglandins and thromboxane A2. The inhibition of thromboxane A2, a potent vasoconstrictor and promoter of platelet aggregation, is crucial for its antithrombotic effects. The endothelial cells lining the blood vessels play a pivotal role in maintaining vascular homeostasis. Aspirin’s ability to inhibit COX-1 reduces the production of pro-inflammatory prostaglandins, leading to a decrease in endothelial inflammation. This anti-inflammatory action is vital in preventing endothelial dysfunction, which is a precursor to atherosclerosis and other cardiovascular diseases. Aspirin also enhances endothelial nitric oxide (NO) production. NO is a vasodilator that helps maintain vascular tone and inhibits platelet aggregation and leukocyte adhesion to the endothelium. By promoting NO bioavailability, aspirin helps improve endothelial function and reduce oxidative stress, a major factor in endothelial damage and cardiovascular disease progression. Furthermore, aspirin influences endothelial progenitor cells (EPCs), which are essential for endothelial repair and regeneration. Studies have shown that low-dose aspirin therapy can increase the number of circulating EPCs, thereby enhancing the repair of damaged endothelium and contributing to vascular health. Additionally, aspirin’s effects on the endothelial expression of adhesion molecules, such as vascular cell adhesion molecule-1 (VCAM-1) and intercellular adhesion molecule-1 (ICAM-1), are significant. By reducing the expression of these molecules, aspirin decreases the adhesion of leukocytes to the endothelium, mitigating the inflammatory response and further protecting against atherosclerosis. In summary, aspirin modulates endothelial biology through its anti-inflammatory properties, enhancement of nitric oxide production, promotion of endothelial repair, and reduction in pro-thrombotic and pro-inflammatory factors. These combined actions contribute to its protective effects on the cardiovascular system, making it a cornerstone therapy in preventing and managing cardiovascular diseases.

P2Y12 inhibitors block the P2Y12 receptor on platelets, preventing adenosine diphosphate (ADP) from binding and activating platelets. By impairing platelet activation and aggregation, these agents reduce the risk of arterial thrombosis and subsequent cardiac events. P2Y12 blockers, such as clopidogrel, prasugrel, and ticagrelor, target the P2Y12 receptor on platelets, which plays a key role in platelet activation and aggregation. By inhibiting this receptor, these drugs prevent adenosine diphosphate (ADP) from binding to P2Y12, thereby reducing platelet aggregation and thrombus formation. This antiplatelet effect indirectly benefits endothelial biology by decreasing the risk of endothelial injury and inflammation associated with thrombosis. Furthermore, P2Y12 blockade has been shown to enhance endothelial nitric oxide (NO) production and improve endothelial function, contributing to vasodilation and vascular health. These agents also reduce the expression of endothelial adhesion molecules, such as VCAM-1 and ICAM-1, diminishing leukocyte adhesion and inflammation within the vessel wall. Through these mechanisms, P2Y12 blockers support endothelial integrity, reduce oxidative stress, and help maintain vascular homeostasis, thus playing a crucial role in the prevention and treatment of cardiovascular diseases.

Additionally, anticoagulants such as warfarin, direct oral anticoagulants (DOACs) like rivaroxaban and apixaban, and heparins (unfractionated heparin and low molecular weight heparin) play a crucial role in CAD management. These medications interfere with the coagulation cascade, preventing fibrin formation, which is essential for stabilizing clots. Warfarin inhibits vitamin K-dependent clotting factors, while DOACs directly inhibit specific clotting enzymes like factor Xa or thrombin. Heparins activate antithrombin III, enhancing its inhibitory effect on thrombin and factor Xa. By disrupting the coagulation process, anticoagulants reduce the formation and growth of thrombi, thus helping to maintain coronary artery patency and mitigate the risk of acute coronary syndromes [30].

When considering heparin anticoagulation, it is advisable to begin with a single intravenous bolus of unfractionated heparin (UFH) before undergoing percutaneous coronary intervention (PCI). UFH is preferred over low molecular weight heparin (LMWH) due to its anticoagulant effect being easily measurable with a simple point-of-care test (the activated clotting time), its shorter half-life (1–2 vs. 4–5 h), and the availability of protamine sulfate as an antidote. Alongside UFH, individuals with hemophilia (PWH) typically undergo dual antiplatelet therapy with aspirin and clopidogrel, following general guideline recommendations and evidence from published case series. We suggest administering a loading dose of 325 mg aspirin and 600 mg clopidogrel before PCI. Following this, dual antiplatelet therapy post-PCI is necessary to prevent stent thrombosis, despite the increased bleeding risk associated with combining the inherited coagulation defect with drug-induced platelet dysfunction [28].

In a French registry documenting long-term antithrombotic treatments in PWH with cardiovascular disease (CVD), bleeding events were more frequent in patients treated with antiplatelet medications compared to those without. Therefore, to minimize bleeding risk, we advise limiting dual antiplatelet therapy (aspirin 80–100 mg daily and clopidogrel 75 mg daily) to the shortest duration, specifically during the 4 weeks following stent deployment. Long-term single therapy with low-dose aspirin 80–100 mg daily is recommended as secondary antiplatelet prophylaxis post-PCI and stent deployment in PWH. Although prasugrel and ticagrelor have shown greater efficacy than clopidogrel in reducing deaths and major cardiovascular events, their use for dual antiplatelet therapy is discouraged due to the higher bleeding risk. Limited experience with glycoprotein IIb/IIIa inhibitors in PWH exists, but their use is discouraged due to their association with a particularly elevated bleeding risk [31].

While recent AHA/ACC guideline revisions have permitted the early use of ticagrelor and prasugrel in ACS treatment, extrapolating this to the PWH population is challenging due to bleeding risks. PWH should consistently take a proton pump inhibitor alongside dual antiplatelet therapy to mitigate the risk of gastrointestinal bleeding. Furthermore, glycoprotein IIb/IIIa inhibitors should be reserved for patients with high thrombus burden during coronary angiography or in cases of slow or no-reflow or thrombotic complications. Cayla et al. recommend daily aspirin without prophylaxis, particularly in severe disease cases due to the significantly higher bleeding risk. If bleeding frequency rises, aspirin should be discontinued. In a prospective study, Tuinenburg et al. suggested extending dual antiplatelet therapy without clotting factors in patients with a residual clotting factor of 25% or higher [21,32,33,34]—Table 3.

### 4.6. Major Bleeding Complications during Follow-Up

A prospective case–control study, named COCHE, investigating cardiovascular disease (CVD) management and follow-up in people with hemophilia (PWHs), was conducted in France from 2011 to 2018. The study included a total of 68 PWHs, with a median age of 65 years (ranging from 39 to 89). Among them, 48 had mild hemophilia, 10 had moderate hemophilia, and 10 had severe hemophilia. The cohort comprised 50 individuals with acute coronary syndrome, 17 with atrial fibrillation, and 1 with both conditions. Throughout the follow-up period, the COCHE group experienced a significantly higher incidence of major bleeding events compared to the control group. Major bleeding-free survival curves diverged significantly between the two groups, including among patients with mild hemophilia [17,22].

Bleeding events were more frequent among patients receiving antiplatelet therapy (AT) compared to controls, regardless of hemophilia severity or specific antithrombotic medication. Hemarthrosis and hematoma were the primary bleeding manifestations in both groups, with gastrointestinal bleeding (GIB) occurring more frequently in the COCHE group.

Notably, GIB episodes were significantly more prevalent in the COCHE group, particularly during antithrombotic treatment involving antiplatelet drugs. Importantly, none of the patients experiencing GIB received proton-pump inhibitors (PPIs) [35].

According to recommendations from the ADVANCE working group, it is advised to maintain a trough level of 5–15% while on dual antiplatelet therapy (DAPT) following percutaneous coronary intervention (PCI) [22,35].

Among the seventeen patients reported by Fogarty et al., only two encountered bleeding while receiving antiplatelet agents. One patient, diagnosed with mild hemophilia A and experiencing ST-elevation myocardial infarction (STEMI), completed a 6-month course of dual antiplatelet therapy (DAPT) without secondary prophylaxis and suffered severe gastrointestinal bleeding. Another patient, diagnosed with severe hemophilia A and non-STEMI (NSTEMI), underwent medical treatment but required an increase in factor VIII (FVIII) replacement after 2 months of single-agent aspirin due to nose bleeding and excessive bruising. Additionally, Fefer et al. observed that all three patients in their study had no significant bleeding during follow-up. There are also reports of instances where patients did not receive FVIII replacement therapy in the setting of acute coronary syndrome (ACS) and received heparin and antithrombotics without bleeding events [20]. Originally, Schutgens et al. advocated for targeting a clotting factor trough level of 30% by administering clotting factor concentrate every 12 h during DAPT. However, subsequent follow-ups suggested that daily dosing was sufficient for patients without severe disease [18,25]. A recent case report by Chang et al. discussed a patient with multivessel PCI and a history of bleeding who was treated with recombinant FVIII, maintaining a trough level above 15 during DAPT. This patient experienced several bleeding episodes at this trough level but remained free of bleeding episodes during a subsequent PCI when maintained at a trough level >20. Despite various recommendations regarding trough goals for replacement therapy, it is believed that aiming for a trough clotting factor level of ≥30% is optimal and associated with fewer bleeding events [29].

## 5. The Impact of Alloantibodies in Hemophilic Patients with CVD

The presence of alloantibodies poses a particularly challenging scenario in the management of acute coronary syndrome in patients with hemophilia. Up to 30% of individuals with severe hemophilia A develop alloantibodies that neutralize the coagulant activity of infused coagulation factors. There is limited guidance available regarding the optimal management approach when patients with inhibitors experience acute coronary syndrome. Due to the ineffective hemostasis achieved with standard replacement therapy, alternative products such as plasma-derived (factor VIII inhibitor bypassing activity, FEIBA) or recombinant (activated factor VII, rFVIIa) agents are necessary [11,36]. These agents contain activated coagulation factors capable of bypassing the inhibitor defect and facilitating hemostasis. However, it is important to note that both bypassing agents carry a potential risk of thrombosis. Therefore, special attention should be given to their use in patients with hemophilia who already have a hypercoagulable state during acute coronary syndrome.

There is still considerable uncertainty regarding the management of coronary artery disease (CAD) in the growing population of patients with factor VIII (FVIII) inhibitors who are receiving prophylaxis with emicizumab.

Emicizumab is a long-acting bispecific monoclonal antibody that functions similarly to activated FVIII. Emicizumab is a bispecific monoclonal antibody designed to treat hemophilia A, particularly in patients with inhibitors to factor VIII. Unlike traditional factor VIII replacement therapies, emicizumab does not directly replace the missing factor. Instead, it mimics the function of activated factor VIII by binding to both factor IXa and factor X, facilitating their proximity and interaction, which is crucial for the activation of factor X to Xa. This activation is a pivotal step in the coagulation cascade, leading to the formation of a fibrin clot. By bypassing the need for factor VIII, emicizumab can effectively promote clotting even in the presence of inhibitors that neutralize exogenously administered factor VIII. Emicizumab also promotes FXa generation on the surface of endothelial cells [36].

Emicizumab is administered subcutaneously, offering a more convenient and less invasive option compared to intravenous factor VIII infusions. It is used both prophylactically and for on-demand treatment in patients with hemophilia A, significantly reducing the frequency of bleeding episodes. The efficacy of emicizumab has been demonstrated in clinical trials, where it has shown a substantial reduction in annualized bleeding rates compared to standard therapies. Additionally, its unique mechanism of action and delivery method provide a valuable alternative for patients who have developed inhibitors to factor VIII, addressing a critical challenge in the management of hemophilia A and improving overall quality of life [37].

Given that patients with hemophilia (PWH) on emicizumab undergoing percutaneous coronary intervention (PCI) cannot be effectively monitored in the laboratory using activated partial thromboplastin time (APTT)-based coagulation factor assays, alternative methods such as bovine chromogenic assays should be employed. These assays are crucial for monitoring and adjusting FVIII levels following concentrate infusion and heparin therapy in this patient population. The role of monitoring emicizumab in patients with hemophilia and cardiovascular disease is multifaceted, aiming to optimize treatment efficacy while minimizing the risk of complications, particularly thrombotic events. In hemophilia management, regular monitoring of emicizumab therapy is essential to ensure that patients receive the correct dose to maintain adequate hemostasis without increasing the risk of thrombosis. Traditional coagulation assays, such as activated partial thromboplastin time (aPTT) and factor VIII activity assays, are not suitable for monitoring emicizumab due to its unique mechanism of action. Instead, global coagulation tests, such as thrombin generation assays (TGAs), are used to evaluate the overall hemostatic potential in patients receiving emicizumab. TGAs can provide insight into the risk of thrombotic complications by measuring the balance between procoagulant and anticoagulant forces in the blood. In patients with hemophilia and concomitant cardiovascular disease, the need for meticulous monitoring is heightened due to the dual risks of bleeding and thrombosis. Cardiovascular disease in these patients often requires anticoagulation or antiplatelet therapy, which can complicate the hemostatic balance. Monitoring emicizumab levels and coagulation parameters helps healthcare providers adjust dosing to prevent bleeding episodes while minimizing the risk of thrombotic events, particularly in the setting of invasive procedures or acute cardiovascular events. Moreover, the interaction between emicizumab and other hemostatic agents, such as bypassing agents used to treat breakthrough bleeds, necessitates careful monitoring. The concomitant use of these agents can significantly increase the risk of thrombotic microangiopathy and other thrombotic complications. By closely monitoring emicizumab levels and coagulation status, clinicians can make informed decisions about the use of additional hemostatic therapies. Lastly, patient-specific factors, such as the presence of inhibitors against factor VIII, renal function, and comorbidities, must be considered when monitoring and adjusting emicizumab therapy. Personalized monitoring strategies ensure that each patient achieves optimal hemostatic control while minimizing the risk of adverse outcomes, ultimately improving the quality of life for patients with hemophilia and cardiovascular disease. In summary, the monitoring of emicizumab in hemophilia patients with cardiovascular disease is critical to balance the delicate interplay between bleeding and thrombosis. Utilizing advanced coagulation assays, assessing drug levels, and considering patient-specific factors are all integral to ensuring safe and effective therapy, highlighting the importance of personalized medicine in this complex patient population [30].

## 6. Coronary Artery Bypass in Hemophilic Patients

The process of cardiac surgery involves coagulation irregularities, leading to heightened risks of bleeding both during and after the operation. These risks stem from factors such as heparinization, surgical trauma, extracorporeal circulation, hypothermia, and increased fibrinolysis. As a result, managing hemostasis while simultaneously administering anticoagulants poses a significant challenge in patients with hemophilia undergoing cardiac surgery [38].

Conducting cardiac surgery on patients with hemophilia presents a formidable challenge in achieving hemostasis. Coagulopathy, particularly induced by total heparinization and extracorporeal circulation, significantly elevates the risk of bleeding. Nevertheless, cardiac surgery remains a viable option for individuals with hemophilia, provided that replacement therapy is meticulously planned with regard to dosage and duration. This planning should be executed under the guidance of a multidisciplinary specialized team, ensuring close clinical and laboratory monitoring and follow-up. Fortunately, the increasing utilization of percutaneous coronary intervention (PCI) in recent years has substantially reduced the necessity for coronary artery bypass grafting (CABG) both in the general population and in patients with hemophilia and coronary artery disease (CAD). Consequently, indications for CABG are now limited to cases of multi-vessel CAD or situations where the PCI revascularization approach is not feasible [39].

Guidance on the most dependable methods for monitoring factor VIII (FVIII) therapy throughout cardiac surgery stages is currently insufficient. However, existing evidence suggests that utilizing thromboelastography (TEG) or thromboelastometry proves effective in determining the ideal dosage and administration frequency of recombinant FVIII (rFVIII). Based on the observations from this case report, once FVIII levels normalize before surgery, TEG can confirm treatment effectiveness, while Hepcon HMS can ensure adequate anticoagulation during cardiopulmonary bypass (CPB) until heparin reversal. In the final intraoperative phase, spanning from heparin reversal to the patient’s departure from the operating room, a combination of TEG and conventional testing optimally guides therapy to achieve normal coagulation and ensure sufficient hemostasis [40]. The International Society on Thrombosis and Hemostasis Scientific and Standardization Committee recommends assessing the viscoelastic properties of clot formation and progression clinically for patients undergoing general surgical procedures. However, their guidelines do not specifically cover the unique circumstances of patients with hemophilia A undergoing cardiac surgery with cardiopulmonary bypass (CPB) [41].

Fogarty et al. noted that all six patients undergoing CABG had unstable angina or NSTEMI and unsuitable findings for PCI on cardiac catheterization. Perioperative hemostatic support included continuous infusion of FIX to maintain a trough level above 100% for one patient, while three received a single bolus of FVIII concentrates followed by continuous infusion. Another patient with mild hemophilia A received desmopressin perioperatively, and a patient with mild hemophilia A and a history of an inhibitor and poor FVIII recovery received recombinant activated FVII. Additionally, adjunctive antifibrinolytic drugs were used in three cases of mild/moderate hemophilia A. Blood components, such as packed red blood cells (pRBCs) and platelets, were administered postoperatively in two cases, including one with chronic hepatitis C virus (HCV) infection [20,41,42].

## 7. Management of Atrial Fibrillation in PWH

The validity of thrombotic (CHA2DS2-VASc) and bleeding (HAS-BLED) risk scores in patients with hemophilia (PWH) is uncertain. The inherent hypocoagulable state complicates the assessment of both thrombotic and hemorrhagic risks using these tools originally developed for the general population. It is unclear whether they may overestimate (CHA2DS2-VASc) or underestimate (HAS-BLED) the corresponding risk in PWH. While the French registry noted more frequent bleeding episodes in PWH with HAS-BLED scores exceeding 3, the tool lacks standardization for use in congenital bleeders. Nonetheless, some advocate for considering oral anticoagulation in PWH with atrial fibrillation (AF) and a CHA2DS2-VASc score of 2 or higher. When oral anticoagulation is initiated, direct oral anticoagulants (DOACs) are preferred over vitamin K antagonists (VKAs) due to a lower risk of cerebral hemorrhage. However, the safety and efficacy of DOACs, as well as optimal dosages, remain unclear in PWH with AF due to insufficiently powered trials. In clinical practice, lower recommended daily doses of DOACs, intended for older patients and those with renal impairment, are often favored, including 220 mg for dabigatran, 15 mg for rivaroxaban, 5 mg for apixaban, and 30 mg for edoxaban [43,44].

In the subset of COCHE patients with atrial fibrillation (AF), HAS-BLED scores of 3 or higher correlated with an elevated risk of bleeding. The HAS-BLED score serves as a crucial tool for gauging baseline hemorrhagic risk before and during antithrombotic therapy in the general populace. In the “Birmingham 3-step therapeutic strategy”, this score constitutes the second step to evaluate bleeding risk and adjust antithrombotic therapy in AF patients. The French registry findings suggest that the HAS-BLED score is applicable to individuals with hemophilia and thus, the “Birmingham 3-step strategy” may be pertinent for this population as well.

For those with hemophilia and nonvalvular AF, anticoagulants (ACs) are recommended if the CHA2DS2-VASc score is 2 or higher, mirroring guidelines for the general population. The transition from vitamin K antagonists (VKAs) to direct oral anticoagulants (DOACs) is ongoing, given DOACs’ documented twofold reduction in fatal bleeding risk for most patients. In the COCHE cohort, individuals with mild hemophilia on AC therapy faced approximately eight times the bleeding risk compared to controls. While major bleeding incidents were linked to VKA use, data on AC therapy were insufficient to compare DOACs and VKAs in hemophilia patients [45,46].

For AF patients with a CHA2DS2-VASc score of 1, the Canadian Cardiovascular Society (CCS) allows for single antiplatelet therapy (SAPT) use, particularly in younger patients, but only when the HAS-BLED score is high, reflecting limited evidence. Recommendations for hemophilia patients often advocate for SAPT in AF cases with a CHA2DS2-VASc score of 1 but may overlook factors like age and bleeding risk scores. In the COCHE study, the slight difference in mean annualized bleeding rates (ABR) between AC and SAPT users (roughly 1.5 times) suggests both may be viable for hemophilia patients with a CHA2DS2-VASc score of 1. However, SAPT is preferable when the HAS-BLED score is 3 or higher, or in patients with severe hemophilia lacking prophylaxis, as per CCS recommendations [31].

To mitigate bleeding risk, continual prophylactic factor concentrate administration is necessary for patients with FVIII/FIX levels below 20%, albeit this approach entails substantial concentrate usage and high costs. Typically, patients with severe hemophilia (clotting factor level < 1%) and AF are not prescribed oral anticoagulants, as their natural decrease in thrombin formation closely resembles that of individuals within the therapeutic range on VKA therapy. Therefore, in patients with FVIII/FIX levels between 1% and 20%, where bleeding risk uncertainties exist, strategies aimed at circumventing the need for long-term anticoagulation should be strongly considered. Non-anticoagulant alternatives for AF management, including cardioversion, catheter ablation, and left atrial appendage closure, have demonstrated efficacy not only in non-hemophilia patients with high bleeding risk (HAS-BLED > 3) but also in those with hemophilia [47].

### Interventional Therapies for AF

Catheter ablation, conducted via a femoral vein approach post-pulmonary vein isolation, aims to disrupt abnormal electrical activity in atrial fibrillation (AF) and restore normal sinus rhythm. In patients with a high risk of bleeding (HAS-BLED ≥ 3), catheter ablation proves as effective as oral anticoagulation in preventing long-term thromboembolic complications but with reduced bleeding risk. A single-center study by van der Valk et al. showed successful maintenance of sinus rhythm in all PWH patients undergoing catheter ablation.

Percutaneous left atrial appendage closure is considered comparable to oral anticoagulation in preventing thromboembolism and cardiac death, especially in PWH with AF. Its goal is to shorten antithrombotic therapy duration and minimize bleeding risk, particularly in those with severe factor deficiency. Despite its potential, limited experience exists in PWH, with few cases identified in the recent literature reviews. Treatment variability exists, but regardless of regimen, antiplatelet therapy for at least 3 months post-procedure, alongside clotting factor prophylaxis is recommended [12,48,49].

## 8. Molecular Insights Regarding Hemophilia and Coronary Artery Disease

Over the past decade, significant progress has been made in understanding the molecular basis of hereditary hemophilias. Early research hinted at mutational heterogeneity, and this has been confirmed by the wide variety of mutations discovered in the coagulation factor VIII gene in hemophilia A and the coagulation factor IX gene in hemophilia B. Both genes are located on the X chromosome: the factor VIII gene is near the end of the long arm at *Xq28*, and the factor IX gene is also on the long arm, closer to the centromere at *Xq27.2*. The substantial genetic distance (0.5 cm) between these genes shows they are not closely linked. Consequently, hemophilia A and B are X-linked recessive disorders, carried by females (karyotype 46) and expressed in males (karyotype 46). Rare instances of female hemophilia can occur, either due to having two defective factor VIII or factor IX genes or because of non-random X chromosome inactivation. The factor VIII and factor IX genes exhibit two types of polymorphisms: single nucleotide polymorphisms (SNPs) and length polymorphisms, also known as variable number tandem repeat sequences (VNTRs) or microsatellites. Despite this, both genes appear to have relatively few polymorphisms. According to the recent human genome sequence data, two randomly chosen haploid genomes differ by 1 base in every 1250 bases on average. Thus, the factor VIII and factor IX genes should theoretically contain approximately 144 and 27 SNPs, respectively. However, the actual number of detected polymorphisms is much lower, which could be due to detection challenges rather than a true scarcity. The human genome sequencing project has identified many more potential polymorphisms than those currently recognized.

Differences in allele frequencies among populations impact linkage studies in hemophilia A. The most diagnostically useful loci may vary with the ethnicity of the family under investigation, which must be considered when planning the investigative strategy.

Linkage disequilibrium has not been studied for all loci, but it is established for some. There is strong, though incomplete, linkage disequilibrium among the intron 18 *BclI*, intron 7 G/A, intron 19 *HindIII*, and intron 25 *BglI RFLPs*. Including these loci in linkage studies could enhance informativeness. Additionally, the intron 18 *BclI* and intron 22 *XbaI* A loci also exhibit linkage disequilibrium, albeit to a lesser extent. Analyzing these loci together increases informativeness significantly more than analyzing them individually. For instance, in whites, combined analysis of *XbaI* A increases informativeness to approximately 65% compared to 49% individually; in Chinese, it increases to 52% from 49%; and in Japanese, it increases to 79% from 48%.

Point mutations, deletions, insertions, and rearrangements/inversions occur in both the factor VIII and factor IX genes. Point mutations, which involve single nucleotide substitutions, are the most common genetic defects, found in about 90% of patients. Deletions are the second most common, occurring in approximately 5–10% of patients. Insertions and rearrangements/inversions are relatively rare among those with hemophilia, except for a specific inversion, known as the intron 22 inversion, which is common in patients with severe hemophilia A [50,51].

Gene therapy is poised to revolutionize the treatment of hemophilias, building on decades of research that have brought us to the brink of approval for two products in Europe and the United States. Valoctocogene roxaparvovec, the first gene therapy for hemophilia A, has received conditional marketing authorization in Europe. Another gene therapy, etranacogene dezaparvovec (AMT-061), for hemophilia B, is also under regulatory review. Additionally, several other gene therapy approaches are in earlier stages of development. These therapies involve a one-time infusion of a genetically modified adeno-associated virus (AAV) designed to deliver either the FVIII or FIX gene to the liver. This leads to the continuous endogenous production and secretion of the missing coagulation factor by hepatocytes, thereby preventing or reducing bleeding episodes. Observations indicate that a single administration of an AAV vector provides sustained clinical benefits for over five years without long-lasting or late toxicities. However, an asymptomatic, self-limiting immune-mediated rise in alanine aminotransferase is commonly observed within the first 12 months after gene transfer, which could potentially eliminate the transduced hepatocytes if not managed with immunosuppressive agents such as corticosteroids [52].

The most effective method for introducing therapeutic genes into target somatic cells, known as transduction, is through the use of modified, naturally occurring viruses as vectors. These viruses are highly evolved to transfer their own DNA, making them ideal for this purpose. Adeno-associated viral (AAV) vectors are currently the preferred choice for gene therapy targeting a range of monogenetic disorders, including hemophilias. Three AAV-based biologics—Luxturna (voretigene neparvovec-rzyl for Leber congenital amaurosis), Zolgensma (onasemnogene abeparvovec-xioi for spinal muscular atrophy), and Glybera (alipogene tiparvovec for familial lipoprotein lipase deficiency)—have received marketing approval, with several other products under regulatory review. AAV vectors are derived from a small, single-stranded DNA-based wild-type parvovirus, which is considered nonpathogenic in humans, weakly immunogenic, and replication deficient, requiring a helper virus (usually adenovirus or herpesvirus) for replication and productive infection. Consequently, AAV vectors have the best safety profile among viral origin vectors [53].

## 9. Conclusions

Managing cardiovascular disease in people with hemophilia is intricate, necessitating a delicate equilibrium between promoting clotting and preventing excessive clot formation. Tailoring cardiovascular care for PWH involves assessing individual factors such as bleeding risk, clotting protection, cardiovascular health, severity of factor deficiency, and bleeding tendencies. Generally, treatment decisions for PWH should mirror those for individuals without hemophilia, provided that factor deficiencies are effectively corrected through tailored replacement therapy. Achieving optimal treatment outcomes and minimizing bleeding risks requires close collaboration among hematologists and other specialists from specialized treatment centers, including cardiologists, radiologists, and surgeons, all of whom play crucial roles in managing cardiovascular issues in PWH.

## Figures and Tables

**Table 1:** This table provides a clear summary of the number of studies at each step of the process, from the initial PubMed results to the final selected studies meeting the specified criteria.

Steps	Number of Remaining Studies
Initial PubMed results	120
Excluded: not written in English or comprising single case reports	59
Selected systematic reviews and meta-analyses plus original articles	61
Excluded: studies focusing on children	1
Final results	60

**Table 2:** Link between hemophilia and CVD.

Aspect	Details
Hemophilia and CVD	Hemophilia believed to offer some protection against CVD due to hypocoagulability. Exact incidence not well known [14].
Mortality from CAD	PWH have lower mortality from CAD compared to age-matched males [14].
Prevalence of CAD	Increases with age in PWH [14].
Age-specific prevalence of CAD	Ranges from 0.05% in PWH under 30 to 15.2% in those aged 60 and older [14,15].
CAD-related mortality	Increased from 2% to 6% between 1972 and 2001 [15].
Standardized Mortality Ratio (SMR) for CAD	Doubled from 1990–1999 to 2000–2007 (0.25 to 0.55) [15].
SMR for myocardial infarction	At 3.0, suggesting higher risk of death among PWH [15].
Factors increasing CVD risk in older PWH	Use of clotting factor concentrates, co-infections (e.g., HIV on antiretroviral therapy), hypertension, diabetes, obesity, lack of physical activity, chronic renal disease [16,17].
Common CVD risk factors in PWH	Hypertension, smoking, obesity, diabetes mellitus, dyslipidemia, family history, antiretroviral therapy [16,17].
Cardiovascular events in PWH	Acute coronary syndrome, cerebrovascular ischemic disease, atrial fibrillation (AF) [16,17,18].
Prevalence of CVD in PWH compared to non-hemophilia	CAD, stroke, and myocardial infarction about twice as high in PWH compared to non-hemophilia males [16,17,18].
Prevalence of CVD in PWH compared to general males	Increased prevalence compared to the general male population [17,18,19].
Atherothrombotic events in PWH	Occur at a younger age. Major associated risk factors: chronic obstructive pulmonary disease, hypertension, hyperlipidemia [18,19].

**Table 3:** Preferred treatment in patients with hemophilia and CVD.

Type of CAD	Choice of Treatment
Stable Coronary Artery Disease	- Long-term low-dose aspirin (≤100 mg/day) - Clotting factor prophylaxis for severe disease - Important for ‘heavy bleeders’ or those with high-risk comorbidities
Acute Coronary Syndromes	- Initial ACS management generally unchanged - Adjustments: withhold/delay aspirin, lower LMWH dose - Hemostatic support with FVIII or FIX - Maintain peak clotting factor levels >80% post-PCI
Arterial Access in PCI	- Radial access preferred to lower bleeding risk - Avoid femoral access due to higher bleeding risk
Best Type of Stents for PWH	- Bare metal stents (BMS) for shorter antiplatelet therapy - Second-generation drug-eluted stents (DES) preferred with one month of DAPT
Antithrombotic Treatment	- Combination of platelet inhibitors and anticoagulants - UFH preferred over LMWH for easier monitoring

This table provides a clear and concise overview of the important considerations and preferred practices in the management of CAD in people with hemophilia. List of abbreviations: ACS—acute coronary syndrome; LMWH—low molecular dose heparin; PCI—percutaneous intervention; PWH—patients with hemophilia; DAPT—double.
